# Effective Interactions and Dynamical Properties of Thermoresponsive Hollow Microgels

**DOI:** 10.1021/acs.macromol.6c01002

**Published:** 2026-07-27

**Authors:** Leah Rank, Angel J. Moreno, Emanuela Zaccarelli

**Affiliations:** † Department of Physics, 615841 Sapienza University of Rome, Piazzale Aldo Moro 2, 00185 Roma, Italy; ‡ CNR Institute of Complex Systems, Uos Sapienza, Piazzale Aldo Moro 2, 00185 Roma, Italy; § Centro de Física de Materiales (CFM-MPC), CSIC-UPV/EHU, P Manuel Lardizabal 5, Donostia E-20018, Spain

## Abstract

Hollow microgels—polymer network shells—are soft colloids whose collective behavior is governed by their intrinsic network structure, their unique topology, and solvent quality. Here, we investigate the effective interactions and the dynamics of hollow microgel suspensions at different temperatures just below the volume phase transition. Despite the particles becoming mechanically stiffer as solvent quality worsens, the extracted effective interactions appear to be softer with increasing temperature. This counterintuitive behavior results from a decrease in monomer repulsion, consistent with a Hertzian description. Bulk simulations show that an effective fluid representation is very accurate in describing the structure of monomer-resolved systems, quantified by their radial distribution function, in an extended range of packing fractions well beyond random close packing. Instead, dynamical observables, such as self-diffusion coefficient and collective relaxation times, are only qualitatively captured, with a systematically faster dynamics observed in coarse-grained models due to the absence of internal degrees of freedom. These results identify hollow microgels as ideal elastic shells, interacting as Hertzian particles, well beyond the two-body regime, as opposed to standard microgels, where such an effective description is only valid in the fluid region of the phase diagram.

## Graphical abstract

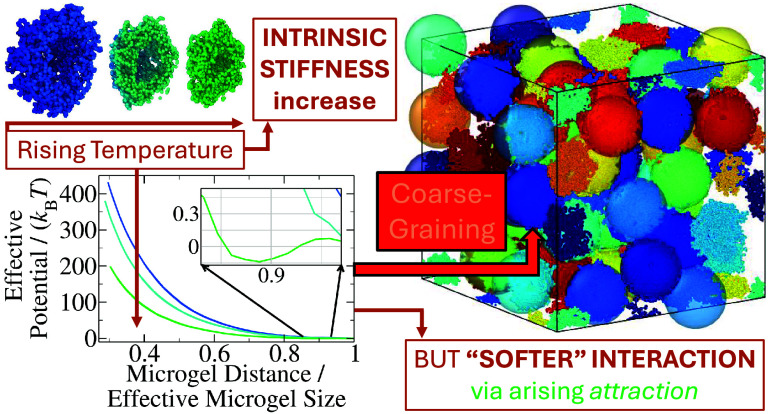

## 1 Introduction

Colloidal suspensions of soft particles are invaluable model systems for exploring fundamental aspects of condensed matter physics, owing to their tunable interactions, internal flexibility, and rich phase behavior.^1–8^ Among soft colloids, *microgels*—cross-linked polymer networks that can reversibly swell or deswell in response to external stimuli—represent an especially versatile class.^9^ Their ability to deform and change size with an external control parameter, such as temperature or pH, provides a suitable platform for studying the interplay between elasticity, effective interactions, and collective behavior.^10–16^ In particular, thermoresponsive poly­(*N*-isopropylacrylamide) (pNIPAM) microgels, which exhibit a well-defined volume phase transition (VPT) temperature near 32 °C,^17^ have become benchmark systems for understanding how softness and deformability affect colloidal assembly and dynamics.^18–21^

Recent works have established a realistic monomer-resolved model of microgels, whose effective interactions,^2^ structural and dynamic properties in bulk^20^ have been recently studied. However, a similar investigation regarding *hollow microgels* has not yet been reported. Hollow microgels consist of a polymeric shell surrounding an internal cavity,^22–24^ obtained by using a sacrificial core in the chemical synthesis. Given their different internal architecture, they display properties that can be markedly different from their regular counterparts. By varying the synthesis parameters, such as the amount of cross-linkers and the width of the elastic shell, it is possible to tune their softness.^25^ In particular, hollow microgels with thin shells and high elasticity—quantified by a large amount of cross-linkers in the network—can undergo large-scale deformations, making them promising not only for encapsulation and release applications,^26,27^ but also as compelling analogues of biological shells such as vesicles or red blood cells.^28–32^ In a recent work, some of us demonstrated that these hollow microgels at high packing fractions spontaneously experience buckling, forming asymmetric, bowl-like shapes due to the competition between crowding and elasticity.^33^ On the other hand, if elasticity is reduced, such buckling instability does not occur. It is important to note that the chosen microgels are realizable in experiments.^25,34^

In the present work, we provide for the first time a thorough characterization of hollow microgels from the dilute and moderately concentrated regimes up to just above the glass transition, also as a function of temperature, and to establish the validity and limitations of an effective pair potential description for structural and dynamic properties. To this aim, we perform large-scale, monomer-resolved Molecular Dynamics simulations of *N*_mgel_ identical microgels for two values of cross-linker concentration *c*. By systematically varying temperature across the good-solvent regime to near-VPT range, we quantify their structural behavior, quantified by their radial distribution functions, and their microscopic dynamics through the mean-squared displacement (MSD), and the interparticle scattering function *F*_inter_(*q, t*), thus characterizing both single-particle and collective relaxation processes.

In parallel, we also provide a theoretical description of bulk suspensions of hollow microgels by evaluating the *effective pair potentials* between two microgels via umbrella sampling simulations and compare them to the Hertzian model, often used to model elastic particles.^2,35^ With the calculated potential, we carry out additional coarse-grained effective-fluid simulations, thus performing a direct comparison of the structural and dynamical behavior of this model with the monomer-resolved systems in an extended range of packing fractions.

Our multiscale analysis reveals intriguing results. First of all, we find that the Hertzian model is able to describe very well the structure of the suspensions for packing fractions even well above random close packing, in stark contrast to regular, nonhollow microgels.^20,36^ Next, given the accuracy obtained for the structure, we interrogate the dynamics of the suspension, performing for the first time such a comparison for monomer-resolved microgels of any kind. We thus find limited validity of the model due to the lack of internal degrees of freedom, which makes the dynamics significantly faster than in monomer-resolved systems. Within this main picture, we also shed light on interesting features related to the temperature behavior. On one hand, we find that the repulsive potential becomes softer with increasing effective temperature, despite the increased stiffness of the microgels, due to the concomitant deswelling. On the other hand, such a deswelling is not solely responsible for the speeding up of the dynamics observed at higher temperatures. Indeed, even when this is taken into account by comparing the data at the same equivalent packing fraction, intermediate temperature simulations are found to be more diffusive, in analogy to what is observed for short-range attractive colloids,^37^ despite the completely different interactions at work. Altogether, the present work bridges elasticity, interactions, and dynamics, thus establishing a comprehensive understanding of how internal softness and cross-linking govern the emergent behavior of hollow microgels in suspension at different temperatures below the Volume Phase Transition.

## 2 Models and Methods

### 2.1 Monomer-Resolved Simulation Details

We generate hollow microgels following the numerical assembly procedure introduced in previous works,^25,38,39^ implemented within the oxDNA simulation package.^40^ The resulting structures consist of a disordered cross-linked polymer network confined to a spherical shell, with an inner and outer assembly radius *Z*_in_ = 29 σ_m_ and *Z*_out_ = 40 σ_m_, respectively, where σ_m_ is the monomer diameter (the unit of length throughout this work). The nominal relative shell thickness is therefore fixed to δ_rel_ = (*Z*_out_ – *Z*_in_)/*Z*_out_ = 0.275, a value that matches the experimental investigation of ref 34. Two different cross-linker concentrations are investigated, *c* = 5% and 10%, corresponding to the molar fraction of cross-linking nodes relative to the total number of monomers *N*_m_ in the network. These values provide the hollow microgels with enough elasticity, ensuring the stability of the hollow architecture, i.e., leaving the cavity intact, across the explored range of temperatures and solvent conditions.^25^

All monomers interact via a standard bead–spring model^41^ that captures the essential polymeric interactions. The excluded-volume repulsion between all pairs of monomers is described by the Weeks–Chandler–Andersen (WCA) potential

1 $$V_{\mathrm{WCA}}\left(r\right)=\{\begin{array}{l} 4\varepsilon \left[{\left(\frac{\sigma_{m}}{r}\right)}^{12}-{\left(\frac{\sigma_{m}}{r}\right)}^{6}\right]+\varepsilon ,,r\leq 2^{1/6}\sigma_{m}\\ 0,,r>2^{1/6}\sigma_{m} \end{array}$$

while directly bonded monomers are additionally connected by a finite extensible nonlinear elastic (FENE) potential

2 $$V_{\mathrm{FENE}}\left(r\right)=-\epsilon k_{F}R_{0}^{2}\ln\left[1-{\left(\frac{r}{R_{0}\sigma_{m}}\right)}^{2}\right],,r<R_{0}\sigma_{m}$$

Here, ϵ sets the energy scale, and all particles have unit mass *m*_m_ = 1. The FENE parameters are fixed to *k*_F_ = 15 and *R*_0_ = 1.5, ensuring bond stability and network elasticity consistent with previous studies.^25,38,39^

To incorporate thermoresponsive behavior, we introduce an additional solvophobic potential,^42,43^ which effectively captures the reduced affinity of the polymer to the solvent upon increasing temperature

3 $$V_{\alpha }\left(r\right)=\{\begin{array}{l} -\varepsilon \alpha ,,r\leq 2^{1/6}\sigma_{m}\\ \frac{1}{2}\varepsilon \alpha \left[\cos\left(\gamma {\left(r/\sigma_{m}\right)}^{2}+\beta \right)-1\right],,2^{1/6}\sigma_{m}<r\leq R_{0}\sigma_{m}\\ 0,,r>R_{0}\sigma_{m} \end{array}$$

with β = 2π – 2.25γ and γ = π/(2.25 – 2^1/3^). The parameter α acts as an effective temperature: α = 0 corresponds to good solvent conditions, while the volume phase transition occurs around α ≈ 0.65.^44,45^ In this work, we explore a range of α values to systematically probe the impact of solvent quality on the equilibrium structure and dynamics of hollow microgels reaching values just below the VPT.

Each microgel contains approximately *N*_m_ ≈ 13000 monomers, a size sufficient to preserve a stable cavity while remaining computationally accessible.^25^ The initially assembled microgel is replicated to build suspensions consisting of *N*_mgel_ = 54 particles in a cubic simulation box of length *l*_box_ with periodic boundary conditions. We perform Molecular Dynamics simulations in the canonical ensemble (NVT), integrating the equations of motion with a leapfrog scheme and using the stochastic velocity-rescaling thermostat^46^ to maintain a constant temperature. The time step is fixed to δt* = 0.002, where time is measured in units of $\sqrt{m_{m}\sigma_{m}^{2}/\epsilon }$ .

To study the dynamics, we focus primarily on the dilute and moderately concentrated regimes, corresponding to packing fractions ζ ≤ 1.0, defined as

4 $$\zeta =\frac{4\pi R_{H,\mathrm{dilute}}^{3}N_{\mathrm{mgel}}}{3l_{\mathrm{box}}^{3}}$$

where *R*_H,dilute_ is the hydrodynamic radius of a single microgel in the dilute limit, evaluated from equilibrium simulations of each cross-linker regime at a given temperature. All systems are equilibrated for 5 × 10^6^ timesteps, followed by a production run of 2 × 10^7^ timesteps, during which structural and dynamical observables are collected.

### 2.2 Calculated Observables

The size and shape of individual hollow microgels are characterized by their radius of gyration

5 $$R_{g}=\sqrt{\frac{1}{N_{m}}\sum_{i=1}^{N_{m}}{\left({\mathrm{r⃗}}_{i}-{\mathrm{r⃗}}_{\mathrm{cm}}\right)}^{2}}$$

and their hydrodynamic radius *R*_H_, computed following refs 47,48 as

6 $$R_{H}=\left\langle 2{\left[\int_{0}^{\infty }\frac{1}{\sqrt{\left(a_{1}^{2}+\theta \right)\left(a_{2}^{2}+\theta \right)\left(a_{3}^{2}+\theta \right)}}d\theta \right]}^{-1}\right\rangle$$

where *a*_1_, *a*_2_, and *a*_3_ are the principal semiaxes of the gyration tensor of the convex hull enclosing the particle.

The internal monomer density profile ρ­(*r*) is obtained as

7 $$\rho \left(r\right)=\left\langle \sum_{i=1}^{N_{m}}\delta \left(\left|{\mathrm{r⃗}}_{i}-{\mathrm{r⃗}}_{\mathrm{cm}}\right|-r\right)\right\rangle$$

averaged over equilibrated configurations. The microgels’ network is analyzed by reporting the scaling of the length of the chains composing the network as a function of the number of monomers in the chain in the Supporting Information (SI) (Figure S1), confirming the expected Flory scaling under all conditions examined in the present work. Additional characterizations of the internal structure of the microgels and of their density profiles are reported in Figures S2–S5.

To analyze the dynamics, we compute the mean-squared displacement (MSD)

8 $$\left\langle \Delta r^{2}\left(t\right)\right\rangle =\frac{1}{N_{\mathrm{mgel}}}\sum_{i=1}^{N_{\mathrm{mgel}}}\left\langle \right|{\mathrm{r⃗}}_{i}\left(t\right)-{\mathrm{r⃗}}_{i}\left(0\right){|}^{2}\rangle$$

the interparticle intermediate scattering function *F*_inter_(*q*_max_,*t*), probing spatial correlations between distinct particles

9 $$F_{\mathrm{inter}}\left(q_{\max},t\right)=\frac{1}{N_{\mathrm{mgel}}}\left\langle \sum_{j=1}^{N_{\mathrm{mgel}}}\sum_{k=1}^{N_{\mathrm{mgel}}}e^{\mathrm{iq}\cdot \left[r_{j}\left(t\right)-r_{k}\left(0\right)\right]}\right\rangle$$

The value |*q⃗*_max_| = *q*_max_ marks the point at which the static structure factor

10 $$S\left(q\right)=\frac{1}{N_{\mathrm{mgel}}}\left\langle \sum_{j=1}^{N_{\mathrm{mgel}}}\sum_{k=1}^{N_{\mathrm{mgel}}}e^{\mathrm{iq}\cdot \left[r_{j}\left(0\right)-r_{k}\left(0\right)\right]}\right\rangle$$

reaches its maximum value at a given packing fraction, roughly marking the size of our microgels. Probing only this value for the scattering functions, we are able to follow the microgels’ center of mass movement.

From the above calculations, we can recover the microgels’ self-diffusion coefficient

11 $$D=\lim_{t\to \infty }\frac{\left\langle \Delta r^{2}\left(t\right)\right\rangle }{6t}$$

as well as the relaxation time τ_inter_, defined as the time at which the interparticle intermediate scattering function decays to 1/*e*, i.e.

12 $$F_{\mathrm{inter}}\left(q_{\max},\tau_{\mathrm{inter}}\right)=\frac{1}{e}$$

### 2.3 Effective Potentials and Elastic Properties

The effective interaction between two identical microgels is determined using umbrella sampling simulations. In this approach, the distance between the microgels’ centers of mass is biased by means of a harmonic potential, which constrains the system around a prescribed equilibrium separation while still allowing thermal fluctuations to be sampled. A series of simulations is performed for different values of the equilibrium distance, resulting in a set of partially overlapping histograms of the interparticle separation. For each sampling window, the probability distribution of finding the microgels at a center-to-center distance *r* is collected. The contribution of the harmonic bias is subsequently removed, and the unbiased distributions from all windows are combined into a single probability function *P*(*r*) via a least-squares method. The effective potential, corresponding to the potential of mean force, is then obtained as

13 $$V_{\mathrm{eff}}\left(r\right)=-k_{B}T\ln P\left(r\right)+C\equiv -k_{B}T\ln g\left(r\right)$$

where *g*(*r*) denotes the radial distribution function and *C* is a constant chosen such that the effective potential vanishes at large separations, *V*_eff_(*r* → ∞) = 0.^49–52^ For our systems, this condition is enforced by setting *V*_eff_(*r*) = 0 for *r* > σ_eff_ for all investigated systems. We have chosen σ_eff_ at the point where we observe a plateau in the calculated effective potential and the microgels no longer feel each other’s presence based on their size *R*_H_.

We further compare the calculated effective potentials to the Hertzian model, fitting the numerical curves to the function

14 $$V_{H}=\frac{2Y\sigma_{\mathrm{eff}}^{3}}{15\left(1-\nu^{2}\right)}{\left(1-\frac{r}{\sigma_{\mathrm{eff}}}\right)}^{5/2}\theta \left(1-\frac{r}{\sigma_{\mathrm{eff}}}\right)$$

where σ_eff_ is the effective Hertzian diameter, as discussed above. For simplicity, we chose σ_eff_ = 2*R*_H_ for all systems except for the highest temperature α = 0.6, since these curves have been fitted disregarding the attractive regions, making the effective size slightly smaller σ_eff_ < 2*R*_H_. Hence, the only fit parameter is the constant *Y*/(1 – ν^2^), being *Y* the Young modulus and ν the Poisson’s ratio. The latter is usually a small number between 0.2 and 0.5,^2^ so that it can be neglected to a first approximation, and we essentially get a rough estimate of the Young modulus of the microgels from this procedure.

The elastic moduli appearing in eq 14 can also be calculated from single microgel simulations using microgel fluctuations as in ref 2 within the phenomenological Mooney–Rivlin theory.^53^ Specifically, we lay a convex hull around each sampled microgel configuration and measure the strain invariants from the convex hull’s gyration tensor. Their histograms yield potentials of mean force (PMFs) from which elasticity information can be extracted, as detailed in the SI, see Figure S6.

### 2.4 Hertzian Effective Fluid Simulations

To compare structural and dynamical properties of the monomer-resolved microgels with the effective fluid representation, we also perform Langevin Dynamics simulations of spheres interacting with the Hertzian potentials established in the previous section for α = 0.0, 0.4, 0.6.

We therefore proceed with the monomer-resolved versus Hertzian simulations by choosing the Langevin friction in order to match the self-diffusion coefficient of the monomer-resolved microgels at sufficiently low packing fraction, namely ζ ∼ 0.2 for α = 0.0, where the MD runs are able to reliably reach a long-time diffusive regime. We do this, knowing that a variation in the short-time properties does not affect the scaling of the long-time dynamics.^54^ The present Hertzian data are compared to existing literature results^55^ in the SI (Figure S7).

To avoid crystallization, we use a polydisperse distribution of *N* = 14000 Hzian particles, uniformly distributed. The radii are drawn to yield an average value of the diameter equal to 1 and a standard deviation of 5%. For simplicity, we use a 7-species mixture, i.e., 2000 particles for each size, efficiently simulated with LAMMPS. We do not find crystallization for all investigated state points.

## 3 Results

### 3.1 Effective Potentials and Elastic Properties

We start by reporting the calculated effective potentials in Figure 1 for both types of hollow microgels studied, with *c* = 5% in (a) and 10% in (b), respectively, at different values of α, i.e., different temperatures, ranging from good solvent conditions α = 0.00 to just below the VPT temperature α ∼ 0.60. For both types of microgels, we find essentially repulsive potentials at low enough α, which move to smaller and smaller onset distances as we increase α due to the shrinking of the microgels with temperature. We thus compare the calculated values with the Hertzian description, whose best fit to the data is also shown in Figure 1. We find that the elastic potential describes the data quite well at intermediate and large distances, in agreement with previous findings for regular^2^ and core–shell^56^ microgels. However, while in those cases strong deviations from the Hertzian behavior appeared already for *V*_eff_ ∼ 10 *k*_B_ *T*, here we find a much more extended range of validity for hollow microgels. In particular, it seems that for the softer microgels at low temperature (α = 0.00), the agreement is satisfactory up to *V*_eff_ ∼ 50 *k*_B_ *T*.

**Figure 1:**
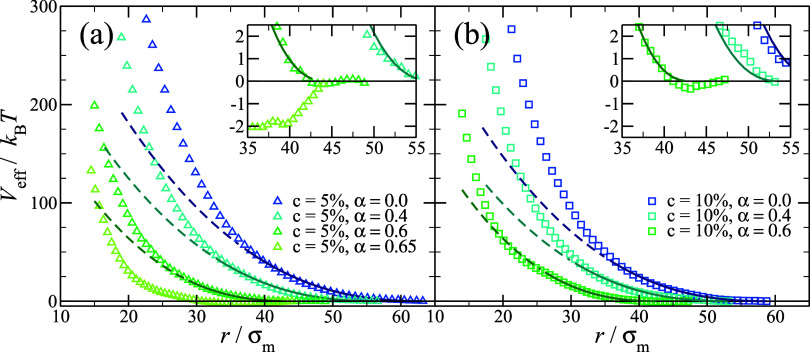
Effective potentials of hollow microgels with cross-linker concentration *c* = 5% (a) and *c* = 10% (b) at different temperatures (α). The colored symbols are the calculated effective potentials, and the solid dark lines underneath refer to the Hertzian fits using the underlying data, while the dashed lines are an extension of the fit functions obtained. The insets in each plot show the zoomed-in potential, highlighting the attractive part at higher temperatures α ≥ 0.6. The Hertzian fits for the α = 0.6 cases have been obtained disregarding the attractive regions (see insets).

As the temperature increases, the agreement with the Hertzian descriptions worsens due to the development of an attractive contribution in the potential, which becomes evident close to the VPT. This is highlighted in the insets of Figure 1(a),(b), where both microgels display the presence of an attractive well in the potential, which becomes more pronounced as we approach *T*_VPT_, i.e., for α = 0.65 for *c* = 5% microgels, where the minimum reaches a value of −2 *k*_B_ *T*. This attraction is expected due to the reduction of solvent quality as the temperature increases. We can still try to impose a Hertzian description on the data, but, obviously, disregarding the attractive contribution, we will not properly capture its full behavior.

It is also instructive to compare the potentials at different temperatures, rescaled by the Hertzian interaction length σ_eff_. This is shown in Figure 2(a) for *c* = 5% microgels, with a behavior that also applies to the stiffer *c* = 10% case. In particular, we find that the potentials become less repulsive as α increases, due to the fact that the repulsion between monomers decreases (by means of an effective attraction). This means that the Hertzian strength *Y*σ_eff_^3^/(1 – ν^2^) decreases with α.

**Figure 2:**
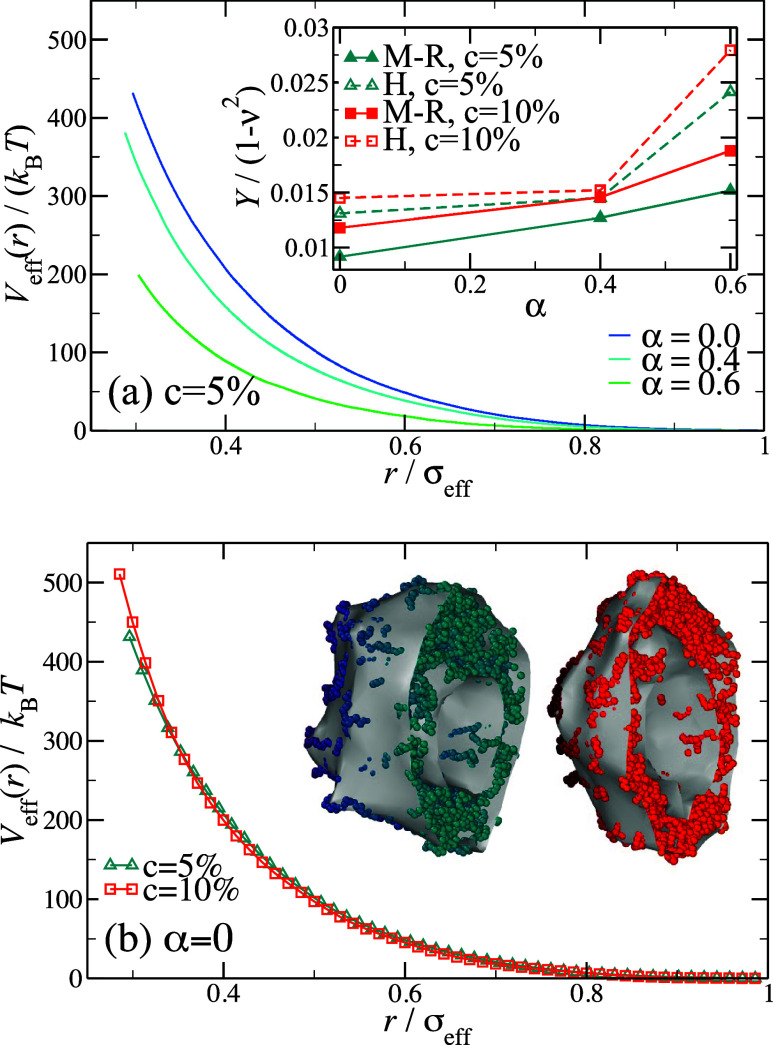
(a) Effective potentials in good solvent for the *c* = 5% microgels, rescaled to their individual effective size σ_eff_. The inset plot shows the elastic moduli ratio *Y*/(1 – ν^2^) for both microgel types (*c* = 5, 10%) at different temperatures for both calculation methods: Mooney–Rivlin (M–R) and Hertzian-fits (H). The results are color-coded to match the curves and snapshots from plot (b), where the curves now refer to the rescaled effective potentials of the two different microgel types in good solvent (α = 0), reporting the cross-linker concentration *c* impact. Snapshots representing each microgel sliced in half and surrounded by their surface mesh are also reported to illustrate the different stiffness between the two studied microgels.

However, we can separately calculate the elastic moduli from single microgel simulations, making use of the Mooney–Rivlin theory.^2^ We find that *Y*/(1 – ν^2^) increases as the temperature rises, as reported in the inset of Figure 2(a). Recalling that *Y*/(1 – ν^2^) ≈ *Y*, because ν is usually ∼0.2–0.3 and cannot exceed 0.5, these results indicate that the microgels get stiffer by collapsing and becoming denser, similarly to regular microgels.^2^ In addition, the *c* = 10% case always lies on top of the *c* = 5% case due to its higher stiffness. While extracting the elastic moduli from the Hertzian fit to the effective potential, also shown in the inset of Figure 2(a), we find that for low enough α the two estimates are in qualitative agreement, but for α = 0.6 the moduli estimated by the Hertzian fits are found to increase too drastically to make up for the shrinkage in size due to this high value of α and removing the attractive portion. As shown above, this is due to the onset of attraction, which makes the Hertzian model no longer reliable, thus giving rise to an apparently very high Young modulus. This could be fixed by adding an attractive term to the Hertzian model to fit the data. However, this is beyond the scope of the present work. Altogether, these results show that the increase in stiffness (*Y*) with α is overpowered by a decrease in effective size (σ_eff_) caused by the increase in temperature, thus resulting in a less repulsive potential approaching the VPT.

Finally, it is also interesting to directly compare the two microgels. For simplicity, we stick to good solvent conditions only (α = 0.0). Despite the increased stiffness of the higher-cross-linked ones, we see in Figure 2(b) that, when the potentials are rescaled by the Hertzian interaction length σ_eff_, the two microgels display an almost identical repulsion, in the whole investigated range. This implies that the reduction in the shell width that is obtained by increasing *c*, as visible in the snapshots also reported in Figure 2(b), is compensated by the increase in the Young modulus, so that the Hertzian strength remains roughly the same. This is an interesting observation, in contrast to the dependence on *c* of the effective potential for nonhollow microgels,^36^ which deserves further investigation by performing additional calculations of effective potentials in the future, varying *c* and shell thickness.

### 3.2 Coarse-Graining

Having calculated the effective potentials and their Hertzian fits, we now aim to coarse-grain the all-monomer simulations using these potentials and also establish the limit of validity of a Hertzian description, with respect to the calculated interactions. We thus perform effective fluid polydisperse simulations where each microgel is represented as a single point particle interacting via the pairwise effective potential, using the Hertzian fits of the numerically calculated potential. In this way, the internal degrees of freedom of the microgels are fully integrated out, and only the effective center-of-mass interactions are retained.

Figure 3 shows the comparison of effective fluid simulations versus all monomer results in terms of the radial distribution function *g*(*r*) for the different cross-linker and temperature cases, covering a wide range of packing fractions. Here, ζ is the packing fraction calculated with respect to the microgels’ hydrodynamic radius in dilute systems, see eq 4. Starting from α = 0.0, we find remarkable agreement between the monomer-resolved and the effective fluid simulations in a range vastly exceeding what was observed for nonhollow microgels,^20^ reported for comparison in the SI Figure S8. In particular, for *c* = 10% the agreement is quantitative up to ζ ∼ 0.8 (see Figure 3(b)), while it even reaches ζ ∼ 1.2 for *c* = 5%, as shown in Figure 3(a). This is well beyond random close packing, reaching a regime where the two-body approximation should no longer be valid.

**Figure 3:**
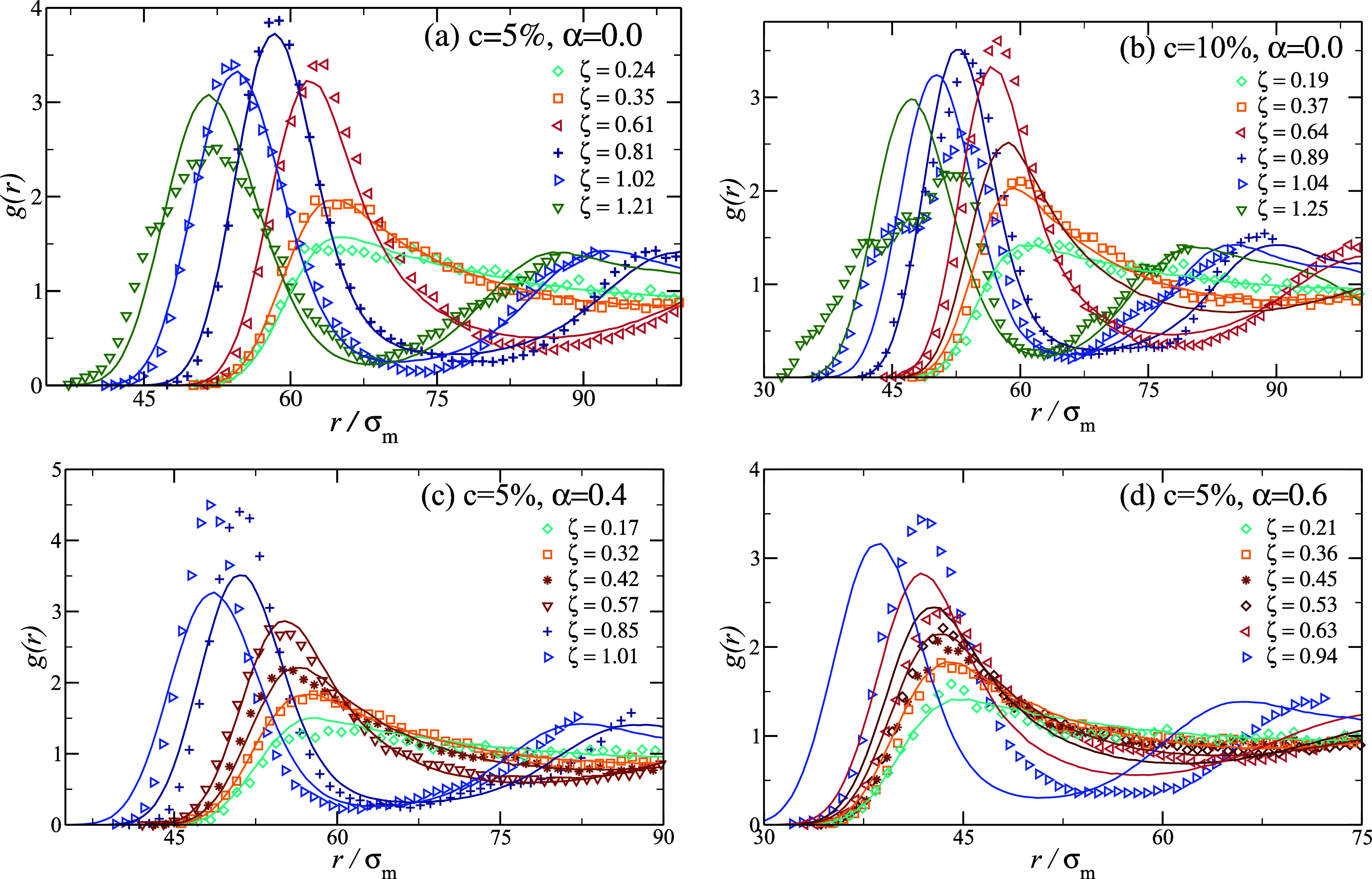
Radial distribution function *g*(*r*) for (a) *c* = 5%, (b) *c* = 10% hollow microgels in good solvent (α = 0.0), (c) *c* = 5% microgels at α = 0.4 and (d) *c* = 5% microgels at α = 0.6. Full lines are effective fluid results, while symbols refer to monomer-resolved simulations.

It is important to connect these findings to our previous work,^33^ which identified the onset of deformation and buckling events right at this point, more evident for *c* = 10%. This is especially visible looking at Figure 3(b), where additional peaks in the *g*(*r*) arise for ζ > 1.0, due to the occurrence of multiple dented configurations of the monomer-resolved microgels, as detailed in ref 33. Hence, we conclude that as long as nonisotropic deformations do not take place, the effective Hertzian model remains a good representation for the static properties of hollow microgels. This extended validity may originate from the fact that in this regime, the microgels are simply trying to close their inner cavity, with a moderate reduction of their size, finally deforming anisotropically when the hollow structure is no longer present. Furthermore, we confirm that as long as this is true, the microgels with different internal elasticity effectively interact with the same Hertzian potential, as shown in Figure 2.

Similar considerations also take place for hollow microgels *c* = 5% at α = 0.4, thus extending the validity of the Hertzian representation in the good solvent regime. Indeed, both the position and height of the first coordination peak, as well as the overall shape of *g*(*r*), are again accurately reproduced as shown in Figure 3(c). However, at higher α, as the Hertzian fit worsens due to the emergence of attraction, deviations between monomer-resolved and Hertzian simulations become evident much earlier, as shown in Figure 3(d). Already below random close packing, we see that the main peak of the Hertzian potential shifts toward the left with respect to the monomer-resolved correlation.

These results suggest that the coarse-grained description is very accurate to describe the structural properties of hollow microgels, contrary to nonhollow ones^20,36^ as shown in Figure S8. Indeed, the main peak position of the *g*(*r*) as a function of ζ is very well-captured by the Hertzian model for both studied *c*, in qualitative contrast with the behavior of regular microgels, where ample discrepancy of the peak position between monomer-resolved simulations and Hertzian model is observed already well below random close packing. These important results thus enable the possibility to perform computationally much more cost-effective bulk simulations for hollow microgels in the low to intermediate ζ regime, even well above random close packing, at low enough α. In what follows, we will attempt to extend the analogy to dynamical properties.

### 3.3 Dynamical Properties of Monomer-Resolved Hollow Microgels and Comparison to the Effective Fluid Representation

We now turn to describe the dynamical properties of the system, first examining the results obtained from the all-monomer simulations. Figure 4(a),(b) show the mean-squared displacement of the centers of mass for the two microgels with different amounts of cross-linkers at α = 0. No significant differences are observed between the two cases. In both systems, the dynamics progressively slow down upon increasing density. We note that the intermediate time regime, usually manifesting as a plateau in the MSD, does not show a completely flat MSD, but rather a long subdiffusive behavior, probably because the centers of masses of the microgels can fluctuate much more than standard spheres due to the internal degrees of freedom of the particles.

**Figure 4:**
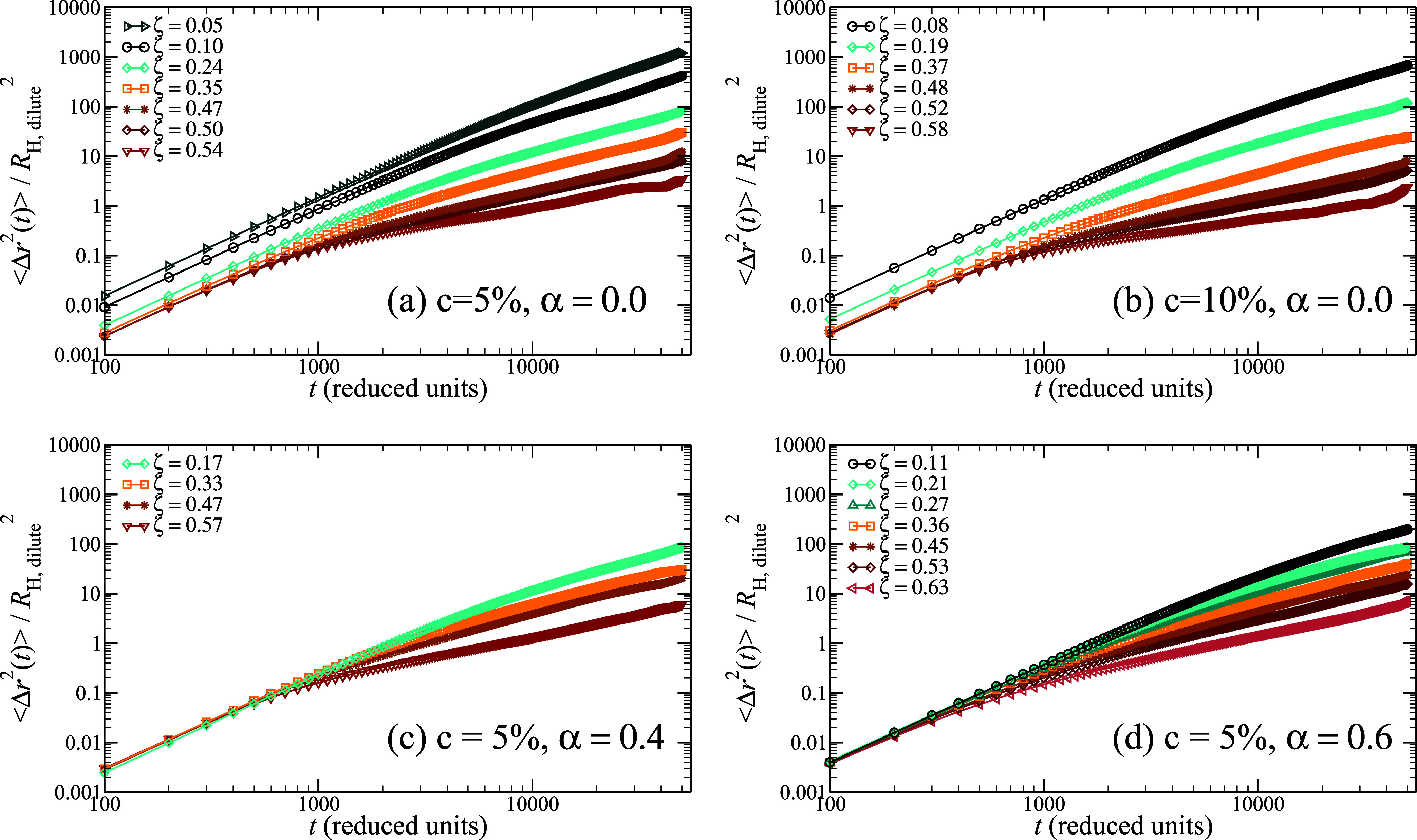
Mean-squared displacement ⟨Δ*r* ^2^(*t*)⟩ for (a) *c* = 5%, (b) *c* = 10% hollow microgels in good solvent (α = 0.0), (c) *c* = 5% microgels at α = 0.4 and (d) *c* = 5% microgels at α = 0.6. In all panels, data are shown for various packing fractions just below the glass transition, where the system can be properly equilibrated. The MSD values have been rescaled by the respective microgel size (for each microgel at each α) in dilute conditions *R*_H, dilute_^2^.

We also report selected MSDs for *c* = 5% hollow microgels in Figure 4(c),(d), respectively for α = 0.4 and 0.6. It is possible to observe that, for comparable packing fractions, the MSD reaches higher values, indicating that the dynamics speed up slightly with increasing α. This is analogous to what is found for hard-sphere colloids with short-range depletion interactions,^37,57^ where the addition of an attractive potential at not too high attraction strengths (i.e., below the VPT temperature for the present case), is able to “melt” the glass. However, it is important to note that despite the analogy with depletion interactions, this result is far from obvious. Indeed, the addition of an attraction is well-known to melt the glass in the case of a colloid with depletion interactions only for sufficiently short-ranged potentials, driven by the use of short polymers as depletants. Here, the interactions at work when raising the temperature are rather different, and due to the worsening of the solvent quality. So, there was no a priori guarantee that a genuine slowing down, already taking into account particle shrinking, would still take place. Although we cannot properly probe the occurrence of dynamical arrest here due to the long computational time needed, the present results indicate that the nearby glass transition shifts to higher packing fractions for intermediate values of α, even after accounting for microgel shrinking. This is clear from examining the MSD for ζ = 0.57 in Figure 4(c) at α = 0.4, which is considerably higher than that of a comparable (and actually even lower) packing fraction (ζ = 0.54) at α = 0 in Figure 4(a).

To gain further insight into the dynamical collective behavior, we also monitor the interparticle intermediate scattering functions *F*_inter_(*q*, *t*) of our monomer-resolved microgels. We first monitor the *q*-dependence of the associated relaxation time (see Figure S9 of the SI), which is found to follow the evolution of the static structure factors. We then focus on the behavior of *F*_inter_(*q*_max_, *t*) evaluated at the wavevector *q*_max_ corresponding to the first peak of the static structure factors, for each studied ζ, thus probing the microgels’ center-of-mass-movement in the nearest-neighbor cage.

Figure 5 reports *F*_inter_(*q*_max_, *t*) for all studied systems at α = 0.0. Interestingly, across all studied state points, we do not observe the characteristic two-step decay typical of the approach to a glass transition. Indeed, no intermediate plateau is detected. Rather than indicating the absence of dynamical slowing down, this behavior may reflect the presence of soft caging in these deformable microgels. While particle motion becomes progressively slower, particle deformability can prevent the formation of the rigid cages that typically give rise to the plateau in the mean-squared displacement and to the corresponding two-step decay in the intermediate scattering function. Future dedicated studies will be needed in order to correctly probe the slow dynamics of these microgels.

**Figure 5:**
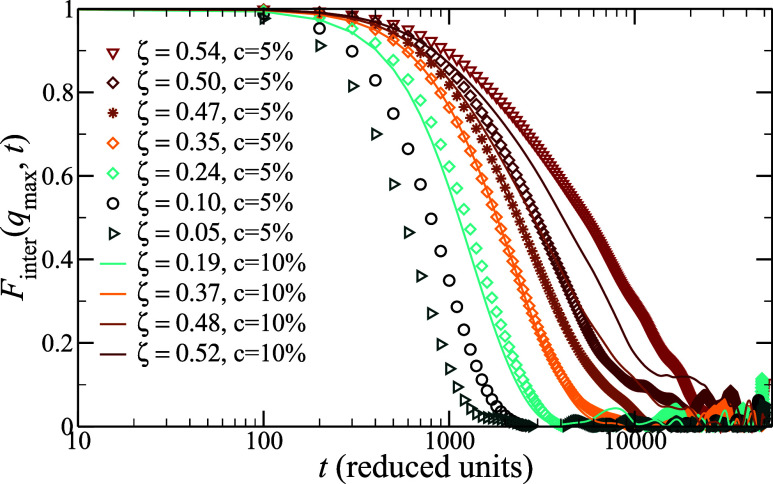
Collective (interparticle) intermediate scattering functions *F*_inter_(*q*_max_, *t*) for suspensions with *c* = 5% and *c* = 10% microgels in good solvent (α = 0.0). All curves are calculated at *q*_max_ denoting the peak position of the static structure factor for hollow microgels with *c* = 5% and *c* = 10% in good solvent (α = 0.0).

To be more quantitative about the distance from the glass transition, we then extract the self-diffusion coefficient *D* from the long-time behavior of the MSD via the Einstein relation. We thus plot *D* versus packing fraction ζ in Figure 6(a). The data confirm that the dynamics are consistently faster with increasing α, particularly at high values of effective packing fraction. This can be explained in terms of the effective potentials, going back to Figure 2(a) for *c* = 5% microgels at different temperatures. Indeed, despite the microgel becoming intrinsically stiffer at higher α due to the increase of the Young modulus, the effective potential becomes softer, thus allowing the microgels to come closer, eventually enabling more movement in equally dense boxes. This is due to the shrinking of the microgel’s size, which appears as a quadratic term in the effective Hertzian potential. These results suggest an analogy with colloids with depletion interactions, where a pocket of liquid states at intermediate temperatures opens up within the glass region,^58,59^ as also observed here for α lower than the VPT. Furthermore, we find that at low packing fractions, as expected from the identical effective potentials, both *c* = 5% and *c* = 10% microgels display very similar values of self-diffusion coefficient for α = 0.0. However, this fails to hold at the largest investigated packing fractions where *c* = 10% microgels become appreciably slower than *c* = 5% ones. This is probably due to their increased stiffness, which makes them closer to an underlying elastic instability, as detected in ref 33, thereby manifesting as an overall slowing down of the dynamics. A qualitatively similar behavior is observed for the collective relaxation time τ_inter_, calculated from eq 12, whose inverse behavior is reported in Figure 6(b). Again, we find slightly faster dynamics at larger α and also for *c* = 5% with respect to *c* = 10% at low α.

**Figure 6:**
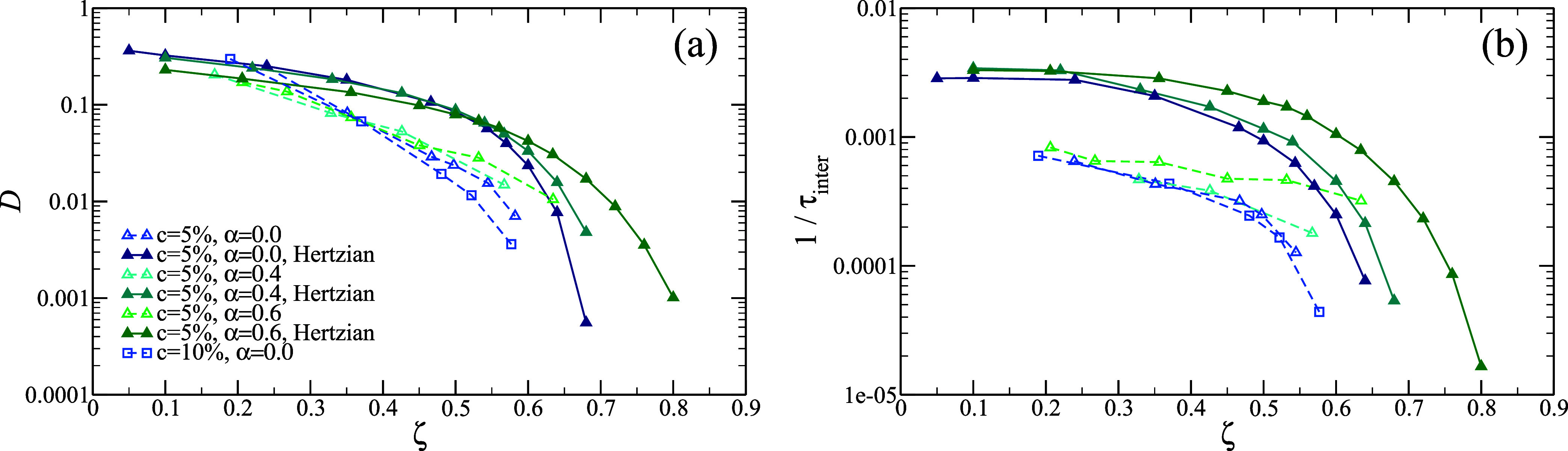
Packing fraction dependence of (a) diffusion coefficient *D* for all monomer-resolved suspensions studied (open symbols, dashed lines) and in comparison with the corresponding effective fluid (Hertzian) results (closed symbols, solid lines) and (b) inverse interparticle collective relaxation times 1/τ_inter_, again for both monomer-resolved together with corresponding Hertzian results. The unit of length of both monomer-resolved simulations and effective fluid is σ_m_.

We now examine the corresponding effective fluid results in more detail. We recall that the friction of the Langevin dynamics was adjusted in order to match the long-time self-diffusion coefficient of the monomer-resolved microgels at the lowest ζ, where the latter could be calculated at α = 0.0, i.e., ζ ∼ 0.2. We then use the same friction for all studied α. Then, the Hertzian data are appropriately rescaled to the monomer-resolved simulations by the corresponding σ_eff_. In this way, we can properly compare the relative decrease of *D* when increasing ζ or α. In particular, while *D* decreases with ζ, we observe that this reduction is smaller at larger α in agreement with the monomer-resolved simulations. Hence, also for the Hertzian fluid, the increase of implicit attraction makes the suspension faster at large enough ζ. However, we observe that the self-diffusion coefficient of the Hertzian results lies consistently above the corresponding monomer data upon increasing ζ. Thus, the effective Hertzian model remains faster at all ζ, since internal degrees of freedom of the monomer-resolved microgels contribute to the slowing down of the dynamics, due to friction and interpenetration. Of course, in the effective fluid representation, these mechanisms are not taken into account, thereby being only qualitatively accurate and consequently leading to an overestimation of the self-diffusion coefficient of about 1 order of magnitude at high ζ. For τ_inter_, again the situation is qualitatively similar, with the only difference that the Hertzian data do not match the monomer-resolved ones even at low ζ. This is due to the fact that we are looking at the nearest-neighbor inverse length *q*_max_, while the friction mapping for the short-time dynamics was made for self-diffusion, corresponding to *q* → 0. Despite these deviations, we conclude that these direct comparisons are rather satisfactory, showing that the effective fluid representation is able to qualitatively describe the dynamics of the hollow microgel suspension.

Finally, we aim to address the question of whether the hollow microgels, in both their full-monomer and effective fluid representation, obey the Stokes–Einstein relationship (SE). In particular, SE states that *D*η ∝ *k*_B_ *T*/*R*_H_, where η is the zero-shear viscosity. While the hydrodynamic radius of soft particles with internal degrees of freedom, such as microgels, can be far from a constant, we previously showed in ref 33 (see Figure 1 of that manuscript) that for ζ < 1.0 the variation of the hydrodynamic radius is negligible for the present systems. Hence, *D*η should still be proportional to a constant at fixed temperature, as in hard sphere colloids.^60^ However, it is computationally prohibitive to calculate η for our monomer-resolved simulations. Hence, we resort to τ_inter_, which should be proportional to η via the shear modulus of the suspension. Assuming that the latter (also difficult to estimate in our simulations) does not change much in the investigated range of packing fractions, the product *D*τ_inter_ has dimension of a squared length, and we can reasonably assume that it should scale as *R*_H_^2^, which, as said above, remains roughly constant for the present system in the investigated dynamical regime. Hence, we report the quantity *D*τ_inter_ as a function of ζ in Figure 7, showing a robust constant behavior for the Hertzian polydisperse fluid, while deviations are observed for the monomer-resolved simulations. In particular, *D*τ_inter_ is found to decrease more rapidly than *R*_H_^2^, since the self-diffusion experiences a decrease of roughly two decades in the investigated ζ-regime, while τ_inter_ does not vary more than about a decade. Since τ_inter_ is found to depend on *q*, in line with the variation of the structure factor (see Figure S9), we do not expect dramatic effects looking at different length scales.

**Figure 7:**
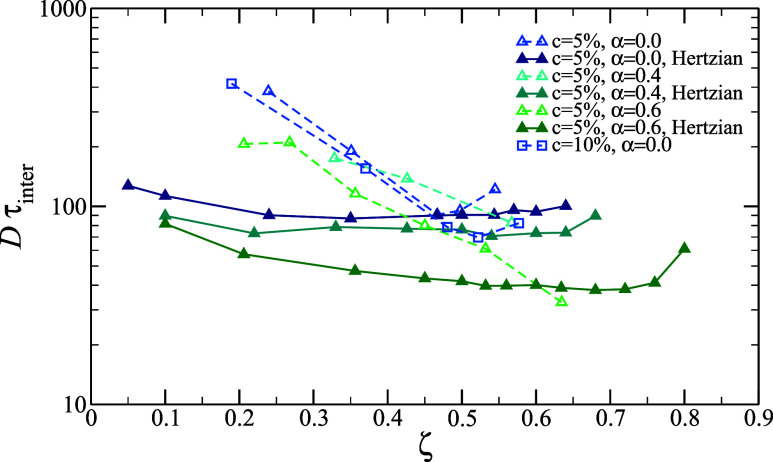
Product *D*τ_inter_ for all studied systems, including both monomer-resolved simulations (open symbols, dashed lines) and Hertzian effective fluid results (closed symbols, full lines) as a function of packing fraction ζ. The unit of length of both monomer-resolved simulations and effective fluid is σ_m_.

It remains an open question to address the physical origin of this decoupling and what happens at larger ζ values, and also to extend these observations to other microgels, including nonhollow ones, as well as to enforce the calculation of the viscosity in the near future to be able to make more quantitative statements on these issues.

## 4 Conclusions

In this work, we have investigated the effective potentials and the dynamics of hollow microgels of varying cross-linker concentrations. These microgels were found to buckle at very high packing fractions, but here we focus on their low- and intermediate-density behavior, focusing on their structural and dynamical properties before the occurrence of a glass transition. We also investigated a few effective temperatures below the Volume Phase Transition, because above it, the suspension becomes highly attractive and equilibration is not possible, except for very low packing fractions, akin to being inside a gas–liquid phase separation for normal liquids. Under these conditions, the microgels would tend to form a gel-like state, which is not the focus of the present work.

First of all, we assessed the effective potentials—calculated via umbrella sampling—of two kinds of hollow microgels differing only in cross-linker concentration (*c* = 5, 10%) at fixed shell thickness. Such a value was selected in our previous work^25^ for a dual reason: on one hand, it reflects the experimental study of Hazra and co-workers,^34^ while on the other hand, despite the microgels being small enough to perform bulk simulations, they still present a clear cavity for both cross-linker concentrations at moderate temperatures.

Hence, for the selected shell thickness, we found that while the microgels become stiffer with increasing temperature as solvent quality decreases, their effective potentials appear to be softer. This counterintuitive behavior is caused by the shrinkage of the microgels, which occurs with increasing temperature and is fully compatible with the Hertzian model, whose repulsive strength scales as *Yσ*_eff_^3^, thereby effectively softening the repulsion. In addition, very close to the VPT, the potential develops an attractive contribution, which eventually leads to a higher apparent Young modulus when trying to fit to a Hertzian model. This signals the breakdown of the Hertzian picture close to the VPT. Intriguingly, comparing the effective potentials of the two microgels with *c* = 5% and *c* = 10%, we find no significant difference when they are rescaled by the respective effective Hertzian lengths.

Importantly, we tested the transferability of these effective potentials to the bulk suspensions by comparing the radial distribution functions obtained from the two sets of simulations: the monomer-resolved system and an effective fluid using the Hertzian fit of the umbrella-sampling calculated potential. Strikingly, we found that the two approaches show remarkable agreement in the calculated *g*(*r*)­s for α = 0.0 up to rather high ζ, even exceeding 1.0 for the lowest cross-linker concentration, and up to random close packing for the highest α tested, suggesting an extended validity of the fluid approach to monitor the structure of the suspension even in very dense conditions. These findings are in stark contrast to what was found for nonhollow microgels, where the range of validity was limited to densities below RCP, as also discussed in the SI. To our knowledge, the present work provides evidence that hollow microgels are the closest representation of 3D Hertzian spheres in a realistic system. This unprecedented agreement for the structural quantities motivated us to further explore the applicability of the Hertzian description also to the dynamics.

Studying the mean-squared displacement for full-monomer microgels in good solvent, first of all, we find a non-negligible difference between the two cross-linker regimes at large enough ζ, despite them being described by rather similar effective interactions. Hence, as microgels pack, the stiffer ones tend to slow down more, as shown from the direct comparison of the self-diffusion coefficient, extracted from the long-time limit of the MSD. Similar results also hold for the collective relaxation times, calculated at the nearest-neighbor peak of the static structure factor. Then, focusing on *c* = 5% and varying the temperature, we also find systematically faster dynamics at higher temperatures, which indicates increased mobility at larger packing fractions. Again, it is worth noting that the definition of effective packing fraction is based on the dilute size of the microgels at the specific temperature, thus already taking into account the deswelling taking place with temperature due to the worsening of solvent conditions. This is thus the result of the softer Hertzian interactions with increasing α.

To be quantitative about the accuracy of the effective fluid representation, we then directly compared *D* and τ_inter_ after appropriately rescaling the former in the dilute regime. We found that the effective fluid is still able to qualitatively describe the dynamical behavior of the system, reproducing the faster dynamics correctly with increasing α. However, we also find that it fails quantitatively by predicting a much less pronounced dynamical slowing down with respect to the monomer-resolved simulations. We attribute this failure to the absence of the internal degrees of freedom of the particles in the effective potential, which contribute more and more at higher densities.

Finally, we observed a decoupling between self-and collective dynamics in the monomer-resolved simulations, which seems to be compatible with a violation of the SE relationship already in the fluid regime for the hollow microgels. This is due to the faster decrease of *D* with respect to τ_inter_ in the full-monomer simulations. However, this does not occur in the Hertzian model, in agreement with previous works.^35^

Given these findings, additional studies will be needed in the future to fully understand this scenario, and to perform a more detailed comparison with nonhollow microgels.^20^ Indeed, this may be useful to assess the role of the central cavity, present in the hollow microgels, which may somehow affect the ability of the microgels’ center of mass (which is most likely found in the empty region) to retain mobility, as opposed to standard microgels with a well-defined core, which is also denser with respect to the outer corona. Similarly, this may shed light on the peculiar absence of a significant two-step decay in the density autocorrelation functions, a feature that is the standard in systems approaching the glass transition. However, this will require very long simulations of the expensive monomer-resolved models and will be left for future work.

In summary, the present study sets a valuable reference assessing the ability of effective coarse-grained models to accurately reproduce structural observables and, qualitatively, the behavior of the dynamics for hollow microgels, so that they can be usefully employed to address fundamental questions in the behavior of large suspensions in a wide range of packing fractions and temperatures below the VPT. Importantly, this applies to a regime limited to low and intermediate packing fractions where large deformations and buckling are still absent. To go one step further, the calculation of many-body potentials by Machine Learning (ML) techniques for anisotropic particles^61^ appears particularly promising, although challenging, and will be investigated in future works.

## Supplementary Material



## Data Availability

Data for this article is available via Zenodo at https://doi.org/10.5281/zenodo.21074539.

## Notes

Microgels’ internal structure is analyzed based on chain-scaling behavior and average monomer density profiles; a more comprehensive discussion of the Mooney–Rivlin theory; the self-diffusion coefficients for the present hollow microgels are placed in the context of previous studies; the radial distribution functions of regular microgels are presented in comparison to the relative Hertzian model; finally the dependence of the relaxation times on the wave vector and its relationship to the structure factor are discussed (PDF)
