# Supplementary material for: Effective Interactions and Dynamical Properties of Thermoresponsive Hollow Microgels
Source: Macromolecules. 2026 Jul 27;59(15):8777–87. doi: 10.1021/acs.macromol.6c01002 (PMC13479960; doi:10.1021/acs.macromol.6c01002)
Supplement: Supplementary file 1 [file ma6c01002_si_001.pdf]

Supporting Information: Effective interactions and dynamical properties of thermoresponsive hollow microgels

Leah Rank,[*,†,‡] Angel J. Moreno,[¶] and Emanuela Zaccarelli[*,‡,†]

†*Department of Physics, Sapienza University of Rome, Piazzale Aldo Moro 2, 00185 Roma, Italy*

‡*CNR Institute of Complex Systems, Uos Sapienza, Piazzale Aldo Moro 2, 00185 Roma, Italy*

¶*Centro de Física de Materiales (CFM-MPC), CSIC-UPV/EHU, P Manuel Lardizabal 5, Donostia E-20018, Spain*

E-mail: leah.rank@uniroma1.it; emanuela.zaccarelli@cnr.it

S1: Inside the microgels

In Figure S1 we plot the average radius of gyration of different chain lengths of the $c = 5\,\%$ microgel as a function of the number of monomers per chain N_l.

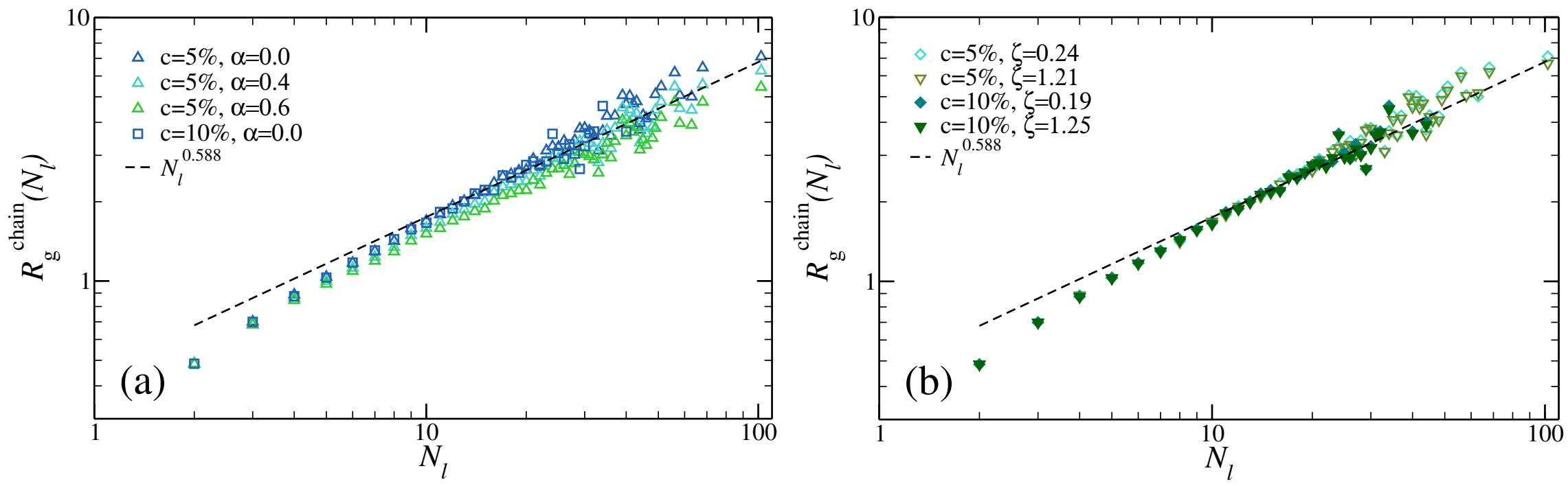

Figure S1: Average radii of gyration $R_{\mathrm{g}}^{\mathrm{chain}}$ of the different chains of length (in terms of number of monomers) N_l (a) in dilute solution at different crosslinker concentration c and temperatures α, respectively (b) at different packing fractions at $\alpha = 0.0$.

We report data for all systems investigated in the manuscript in dilute conditions in Figure S1 (a), as well as results for different packing fractions for both crosslinker concentrations at $\alpha = 0.0$ in panel (b). For long enough chains ($N_l > 10$), the data follow the expected Flory scaling under good-solvent conditions. While a decrease in α continuously decreases the power-law exponent, we find essentially no dependence on ζ up until overlap concentration, so only for $\zeta > 1$ deviations from the Flory exponent start to arise. These results are in agreement with those reported for non-hollow microgels.[1]

Turning now to the average monomer density profiles ρ, we plot the two crosslinker cases in good solvent ($\alpha = 0.0$) at different packing fractions in Figure S2.

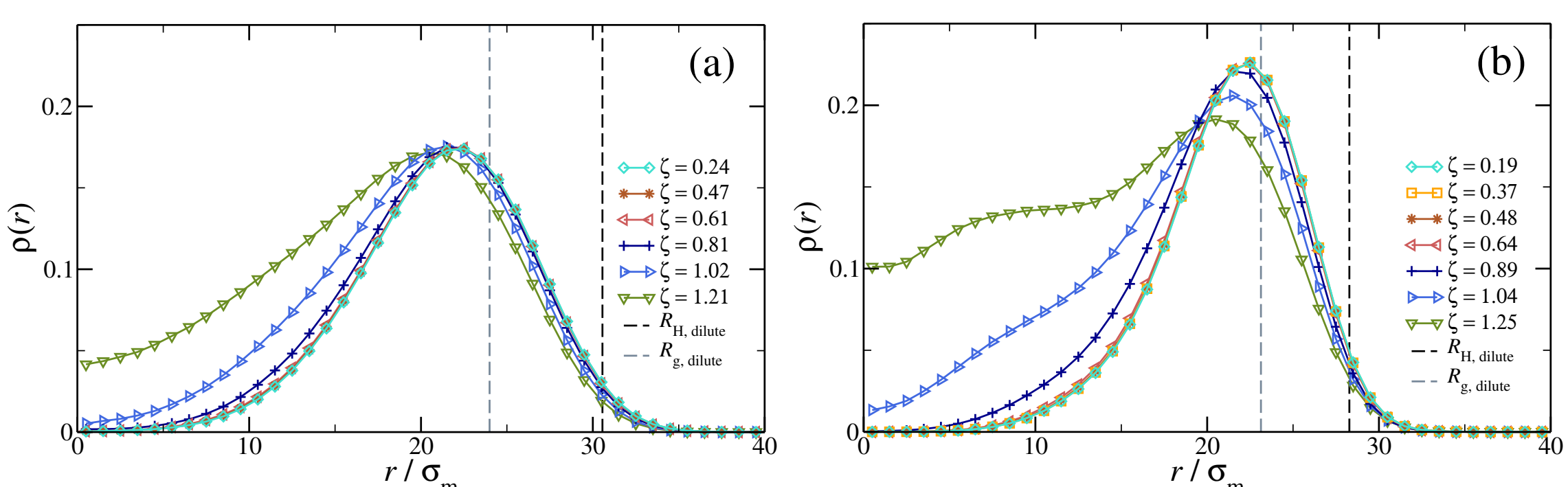

Figure S2: Average monomer density profiles $\rho(r)$ of the diffent microgels types (a) $c = 5\,\%$ and (b) $c = 10\,\%$ at various packing fractions ζ. The vertical dashed lines mark the hydrodynamic radius $R_{\mathrm{H,\,dilute}}$ in black and the radius of gyration $R_{\mathrm{g,\,dilute}}$ in grey for both microgel types in dilute conditions.

In addition, the density profiles in dilute suspensions at the α-values investigated in the manuscript are reported in Figure S3. The curves show that, for both crosslinker cases, the microgel shrinks as α increases but maintains its cavity within the examined density regime. If we were to keep increasing the temperature in dilute conditions, the shells simply collapse onto themselves and become non-hollow while staying spherical, as shown in previous work.[2] To be more quantitative on the dependence of cavity radius and shell thickness as a function of α, we fit the density profiles with the following formula,

$$\rho_{\text{parab}}(r) = \begin{cases} \frac{a}{2}\frac{(r-(R_{\text{in}}-s_{\text{in}}))^2}{s_{\text{in}}^2} & \text{if } R_{\text{in}}-s_{\text{in}} < r \leq R_{\text{in}} \\ \\ a-\frac{a}{2}\frac{(r-(R_{\text{in}}+s_{\text{in}}))^2}{s_{\text{in}}^2} & \text{if } R_{\text{in}} < r \leq R_{\text{in}}+s_{\text{in}} \\ \\ a-\frac{a}{2}\frac{(r-(R_{\text{out}}-s_{\text{out}}))^2}{s_{\text{out}}^2} & \text{if } R_{\text{out}}-s_{\text{out}} < r \leq R_{\text{out}} \\ \\ \frac{a}{2}\frac{(r-(R_{\text{out}}+s_{\text{out}}))^2}{s_{\text{out}}^2} & \text{if } R_{\text{out}} < r \leq R_{\text{out}}+s_{\text{out}} \\ 0 & \text{otherwise.} \end{cases} \tag{S1}$$

This equation essentially consists of four consecutive parabolas with the following defining variables: a, s_{in}, s_{out}, R_{out}, and $R_{\text{in}} = R_{\text{out}} - s_{\text{out}} - W$. Here we set $W = 0$ since the shells are so thin, which yields 4 free fit parameters. From these, we can extract the relative cavity radius $R_{\text{in}}/R_{\text{out}}$ and the effective relative shell thickness $\delta_{\text{eff}} = (R_{\text{out}} - R_{\text{in}})/R_{\text{out}}$.

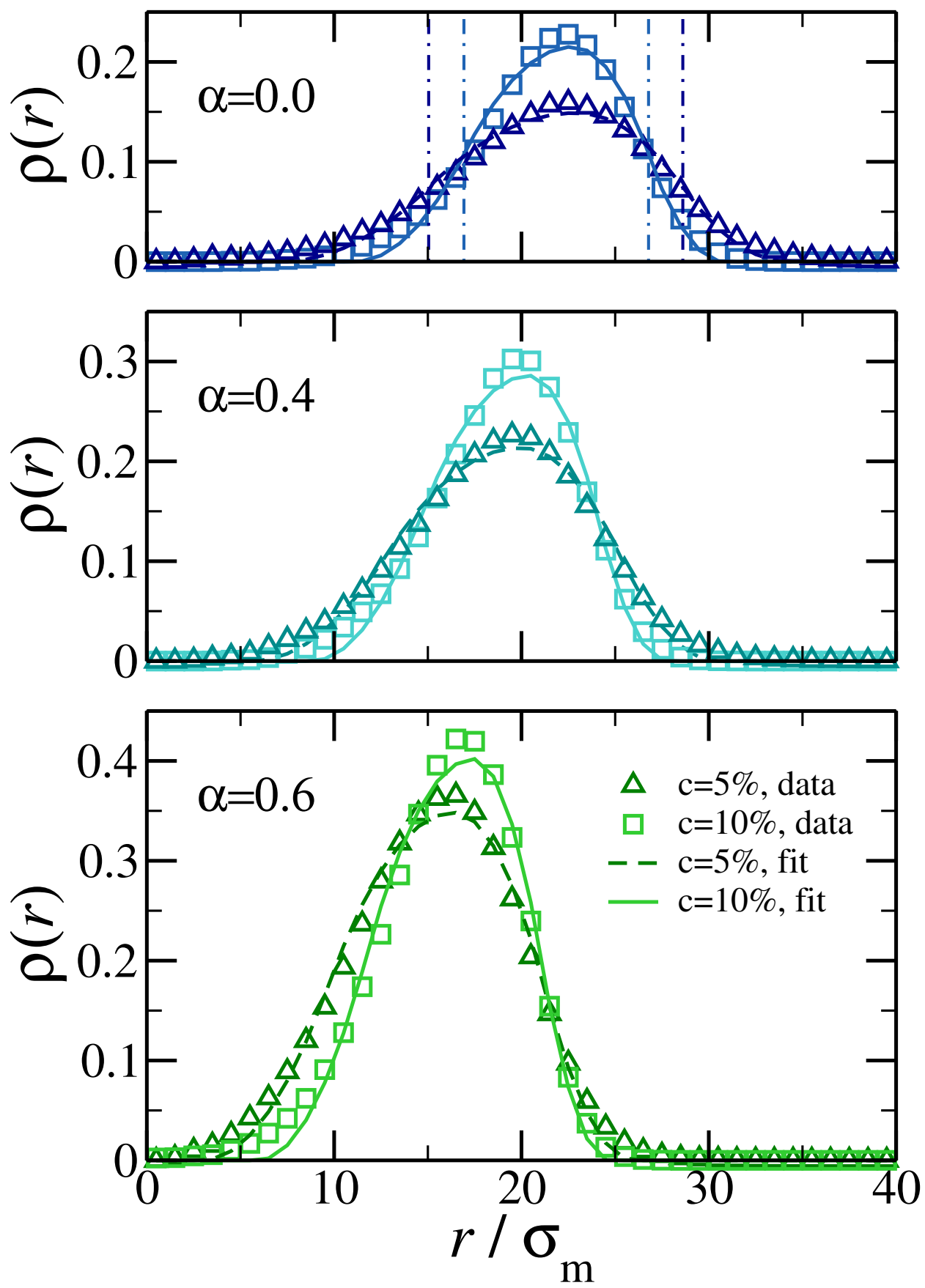

Figure S3: Average monomer density profiles $\rho(r)$ calculated for the microgels with $c = 5\,\%$ and $c = 10\,\%$ in dilute conditions at different temperatures α (symbols) and relative fits using Equation S1. The dash-dotted lines in the first panel point exemplarily to the resulting fit parameters R_{in} and R_{out} for the microgels with $c = 5,\ 10\,\%$, respectively, at $\alpha = 0.0$ in corresponding colours.

The parameters obtained are shown in Fig. S4 as a function of α for the two microgels. We find that the cavity size decreases slightly and the effective shell thickness consequently grows. These results confirm the presence of a cavity for all effective temperatures.

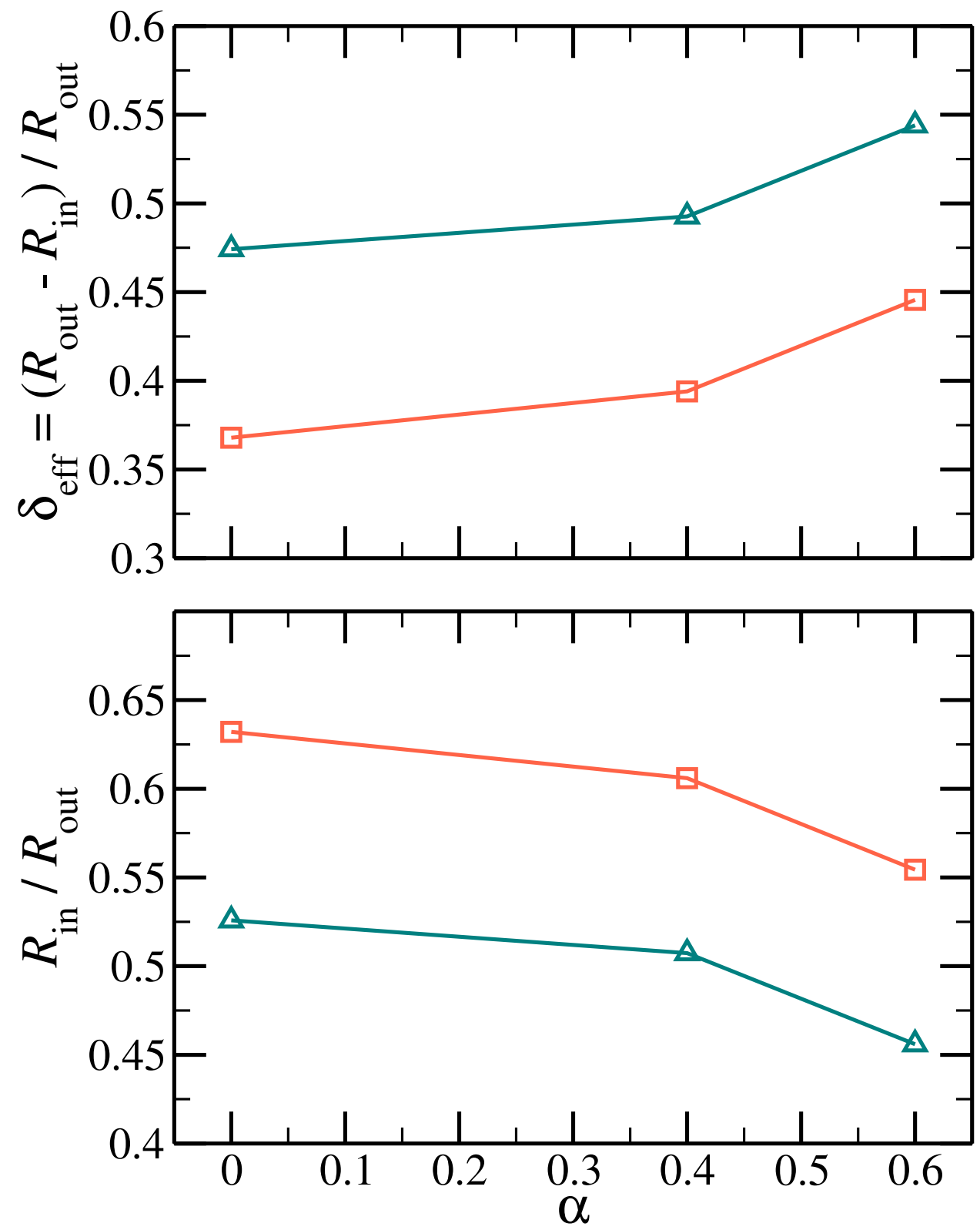

Figure S4: The resulting fit parameters of equation S1 (fits are shown in Figure S3) are plotted against temperature α for both microgels $c = 5$, $10\,\%$. The relative cavity size is given by $R_{\text{in}}/R_{\text{out}}$ and the relative shell thickness is defined by $\delta_{\text{eff}} = (R_{\text{out}} - R_{\text{in}})/R_{\text{out}}$.

To complete the picture, we show the actual values of the different radii for different microgels in different solvents, along with the packing fraction dependence, in Figure S5, emphasizing that there is no change in size in the density regimes we are discussing in the manuscript, *e.g.*, below overlap concentration.

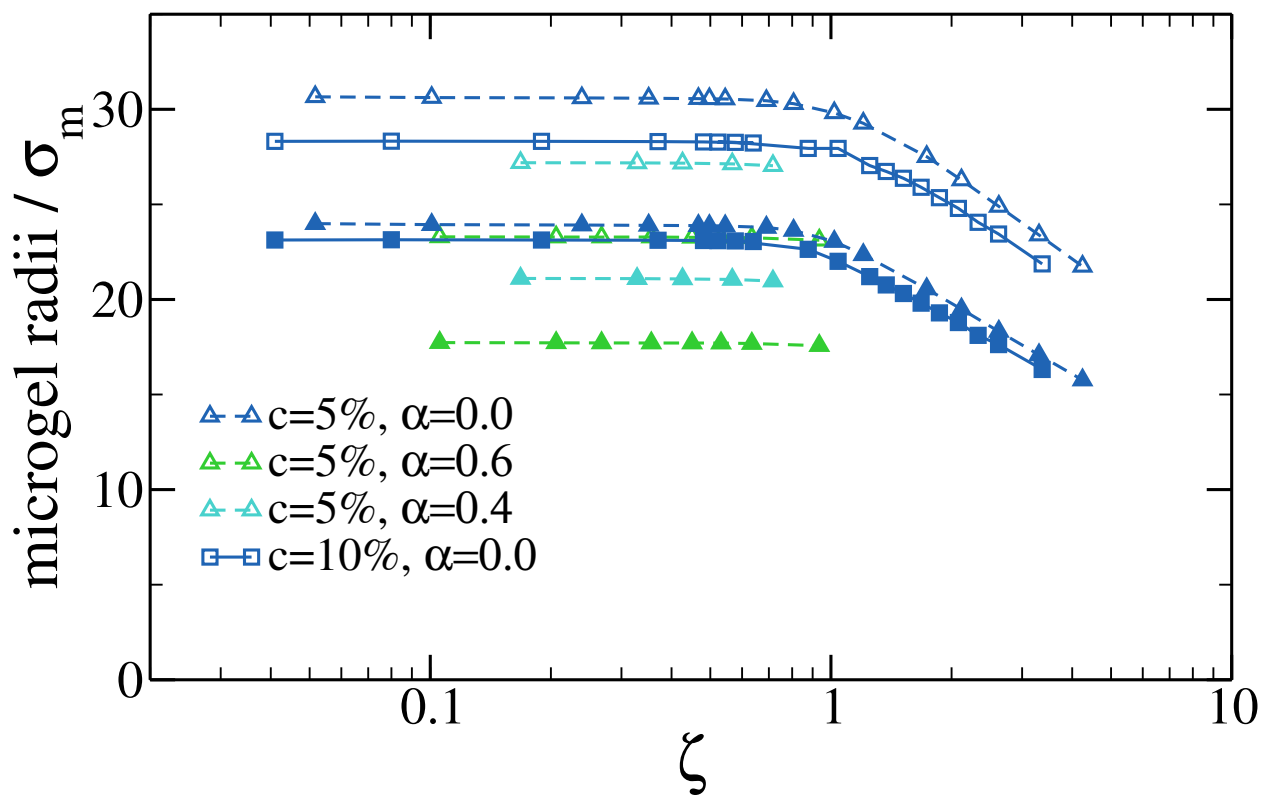

Figure S5: Average microgel radii R_{H} with empty symbols and R_{g} filled symbols of the diffent microgels types $c = 5, 10\,\%$ at different temperatures α as a function of packing fraction ζ.

S2: Elastic moduli calculation - phenomenological Mooney-Rivlin theory

By simulating a single microgel equilibrium trajectory, one can sample the gyration tensor of the microgel's convex hull to extract its eigenvalues λ_1, λ_2, λ_3. The microgel can be approximated as an ellipsoid with semiaxes $a_i = \sqrt{3\lambda_i}$ and the deformation gradient tensor:

$$\mathbf{F} = \begin{bmatrix} \frac{a_1}{\langle a_1 \rangle} & 0 & 0 \\ 0 & \frac{a_2}{\langle a_2 \rangle} & 0 \\ 0 & 0 & \frac{a_3}{\langle a_3 \rangle} \end{bmatrix} \tag{S2}$$

From there, the strain invariants I_1, I_2, and J of the convex hull at each sampled point in time can be obtained:

$$\begin{aligned} J &= \det(\mathbf{F}) \\ I_1 &= \operatorname{tr}(\mathbf{F}^2) J^{-2/3} \\ I_2 &= \frac{1}{2}[\operatorname{tr}^2(\mathbf{F}) - \operatorname{tr}(\mathbf{F}^2)] J^{-4/3} \end{aligned} \tag{S3}$$

Finally, we end up with a list of strain invariants, where the two lists of I_1 and I_2 can be

combined to I - as done by Rovigatti et al. in Reference[3] - and we calculate the distributions of those values $p(I)$, $p(J)$. The logarithm of these distributions leads to the potentials of mean force PMFs $W(I)$ and $W(J)$ reported for a representative case in Figure S6.

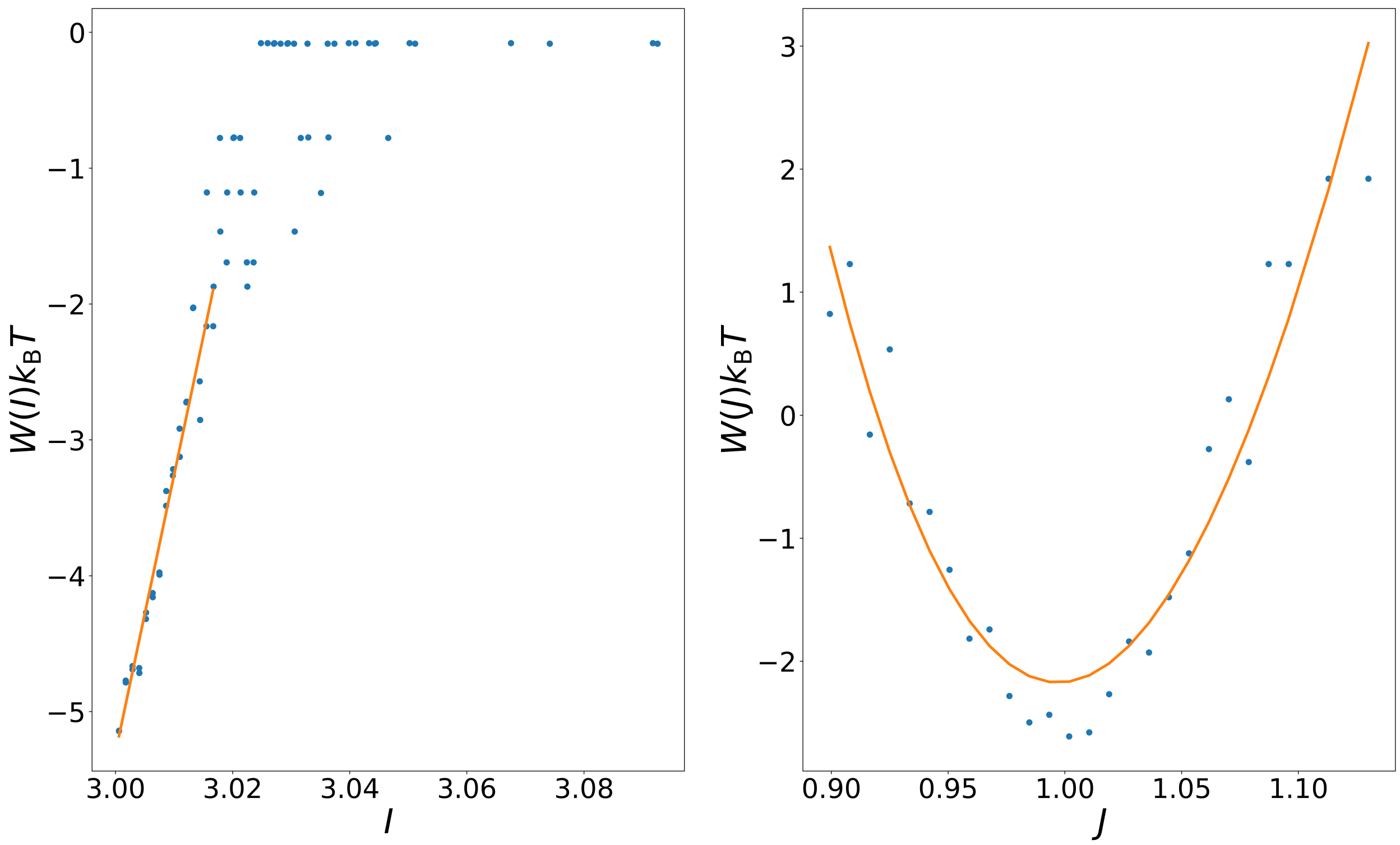

Figure S6: Potentials of mean force (PMFs): $W(I)$ on the left and $W(J)$ on the right of a hollow microgel with $c = 5\,\%$ and implicit temperature $\alpha = 0.0$. The blue dots refer to the calculated data and the orange lines to the resulting fits: $W(I) = M_I I + D_I$, $W(J) = M_J(X_J - J)^2/J + D_J$.

As elaborated in detail by Rovigatti et al.,[3] the two fits - orange lines in Figure S6 - yield the prefactors M_I for $W(I)$ and M_J for $W(J)$, which together with the average volume of the convex hull $V \approx \frac{4}{3}\pi\langle a_1\rangle\langle a_2\rangle\langle a_3\rangle$ eventually give us the bulk modulus K and the shear modulus G of a given microgel:

$$K = \frac{2M_J}{V},\; G = \frac{2M_I}{V}, \tag{S4}$$

and finally[4]

$$Y = 9KG/(3K+G),\; \nu = (3K-2G)/(2(3K+G)). \tag{S5}$$

S3: Comparison to existing Hertzian results

To provide context to existing data, we relate the present data for the Hertzian model explicitly to the results of Pamies et al.[5] in Fig. S7, reporting the self-diffusion coefficients taken from Fig.4 of the 2009 JCP publication, together with the ones of the manuscript for $c = 5\,\%$ microgels as a representative case. Their data are MD simulations and refer to lower repulsive strengths $\epsilon_{\rm H}$ than the ones used in the manuscript. However, the MD data for our work ($\epsilon_{\rm H} = 470 k_{\rm B}T$) are fully in line with Pamies et al.'s data. Moreover, the dynamics of the centers of mass for the monomer-resolved simulations are slower, and this is due, as explained in the manuscript, to the internal friction of the monomers. Thus, to compare the Hertzian dynamics to the monomer-resolved simulations, we employ Langevin Dynamics (LD), adjusting the friction to match the monomer-resolved data at low ζ. As visible from the plot, the use of a different friction only rescales the long-time dynamics without affecting its qualitative behavior, which is well-known in the glass literature.[6] Moreover, the static properties are not affected.

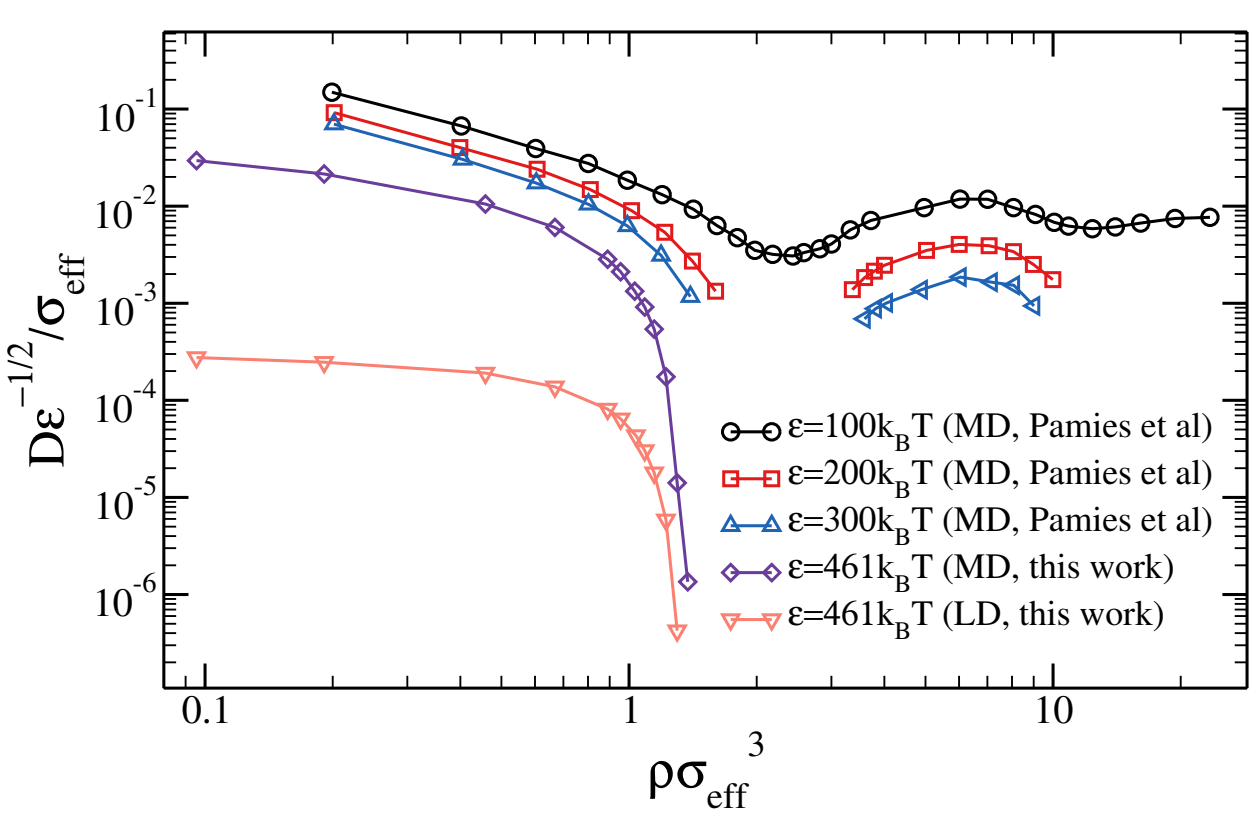

Figure S7: Self-diffusion coefficient (scaled by $\epsilon_{\rm H}^{-1/2}/\sigma_{\rm eff}$) as a function of number density $\rho\sigma_{\rm eff}^3$ taken from Pamies, Cacciuto, and Frenkel[5]) and compared to the present data for the Hertzian model capturing c=5%, $\alpha = 0.0$ microgels, both for MD and for LD simulations.

S4: Radial distribution functions of regular (non-hollow) microgels with $c = 5\,\%$ and comparison with the Hertzian model

In Figure S8, we plot the $g(r)$ results for regular microgels at different packing fractions in a similar fashion as in the manuscript, where symbols represent the monomer-resolved simulations and the lines the Hertzian effective fluid. Data are taken from Ref.[7] and the Hertzian model best representing the numerical data has $\epsilon_{\mathrm{H}} = 470 k_B T$.

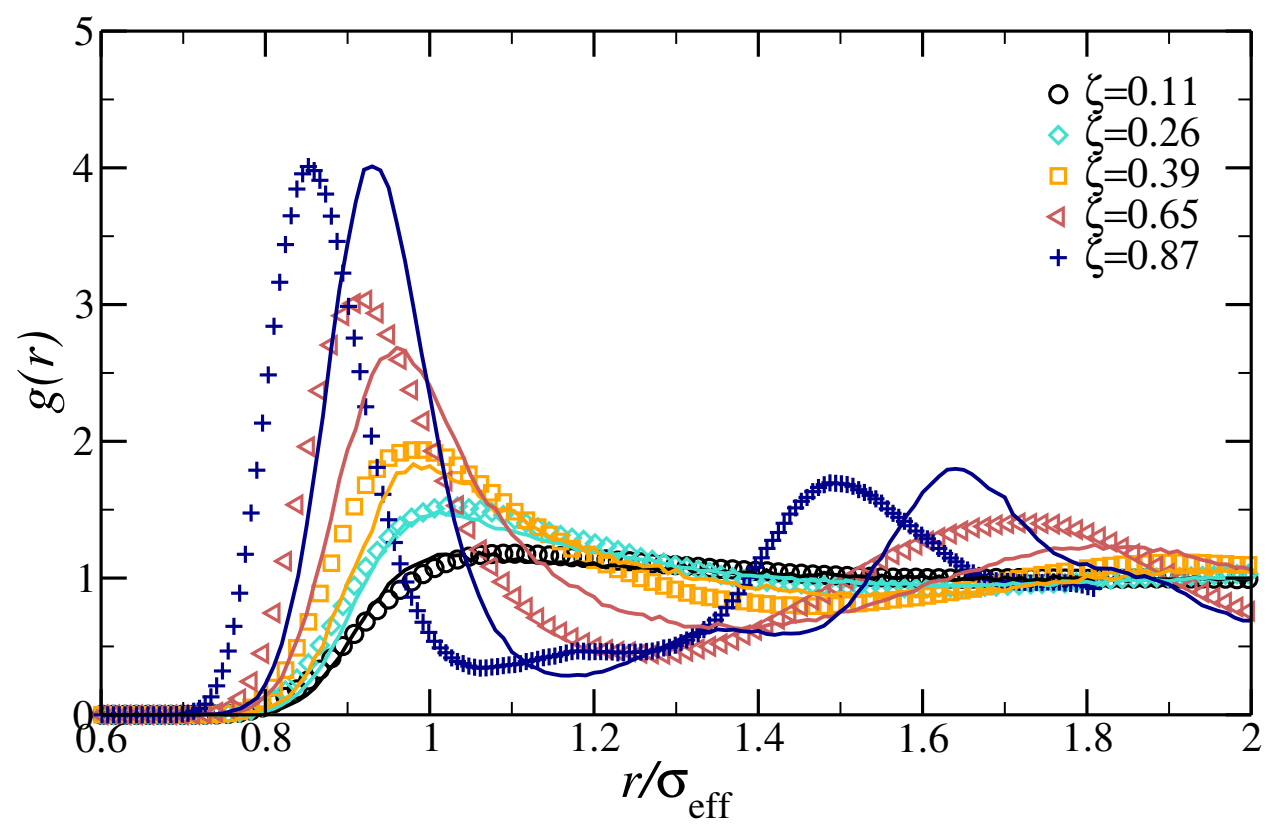

Figure S8: Radial distribution functions $g(r)$ for the monomer-resolved simulations of non-hollow $c = 5\,\%$ microgels in good solvent, taken from Ref.,[7] in comparison to the best Hertzian model ($\epsilon_{\mathrm{H}} = 470 k_B T$) for different packing fractions at $\alpha = 0.0$.

In the regular microgels, the alignment between the two simulation methods already breaks well before random close packing, particularly by a dramatic shift to lower distances of the main peak position in the monomer-resolved simulations with respect to the model. This result is qualitatively different from the behavior observed for hollow microgels, where monomer-resolved simulations, shown in the main text, are found to match the Hertzian counterpart, particularly in the peak position, well beyond overlap concentration.

S5: Relaxation times' dependence on q

The relaxation times $\tau_{\text{inter}}(q)$ at which the inter-particle scattering functions $F_{\text{inter}}(q,t)$ (and their incoherent analogues $\tau_{\text{self}}(q)$ from $F_{\text{self}}(q,t)$) reach $1/e$ obviously depend on the value of q used. Ultimately, we chose $q = q_{\text{max}}$ consistently throughout the paper to obtain all scattering functions and their relaxation times, which is the value at which the structure factor reaches a maximum $S_{\text{max}}(q) = S(q_{\text{max}})$, shown in Figure S9. Simultaneously, the $\tau_{\text{inter}}(q)/\tau_{\text{self}}(q)$-curves in Fig. S9 (a) and (b) present maxima at that same value q_{max}, confirming the validity of our choice.

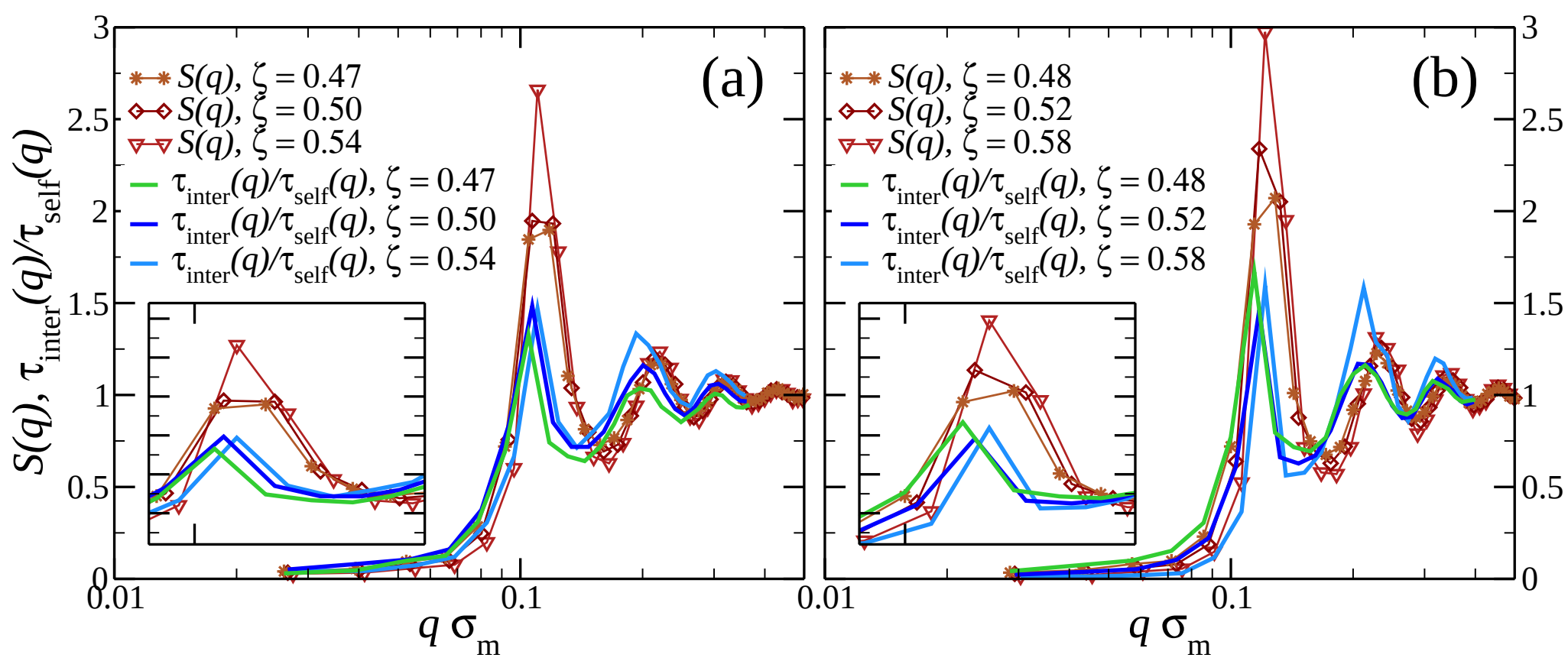

Figure S9: Relaxation time ratio $\tau_{\text{inter}}(q)/\tau_{\text{self}}(q)$ calculated from $F_{\text{inter}}(q,t)$ and $F_{\text{self}}(q,t)$ (the interparticle scattering function and the self analog) evaluated for several values of q and plotted for selected values of intermediate packing fractions ζ together with the structure factor $S(q)$ for the same packing fractions. The left plot (a) shows results for the low crosslinker regime $c = 5\,\%$, while (b) concerns the more elastic microgel ensemble with $c = 10\,\%$ - both in good solvent. The inset plots show the zoomed-in versions to emphasize the maxima overlap of both functions.

References

(1) Marín-Aguilar, S.; Zaccarelli, E. Predicting the structure and swelling of microgels with different crosslinker concentrations by combining machine learning with numerical simulations. *Soft Matter* **2025**, *21*, 8669–8677.

(2) Rank, L.; Zaccarelli, E. Numerical insights on the volume phase transition of thermoresponsive hollow microgels. *Soft Matter* **2025**, *21*, 3979–3990.

(3) Rovigatti, L.; Gnan, N.; Ninarello, A.; Zaccarelli, E. Connecting Elasticity and Effective Interactions of Neutral Microgels: The Validity of the Hertzian Model. *Macromolecules* **2019**, *52*, 4895–4906.

(4) Doghri, I. *Mechanics of Deformable Solids: Linear, Nonlinear, Analytical and Computational Aspects*, 1st ed.; Springer Science & Business Media: Dordrecht, Netherlands, 2013.

(5) Pàmies, J. C.; Cacciuto, A.; Frenkel, D. Phase diagram of Hertzian spheres. *The Journal of chemical physics* **2009**, *131*.

(6) Gleim, T.; Kob, W.; Binder, K. How does the relaxation of a supercooled liquid depend on its microscopic dynamics? *Physical review letters* **1998**, *81*, 4404.

(7) Del Monte, G.; Zaccarelli, E. Numerical study of neutral and charged microgel suspensions: From single-particle to collective behavior. *Physical Review X* **2024**, *14*, 041067.
